# Lack of viable severe acute respiratory coronavirus virus 2 (SARS-CoV-2) among PCR-positive air samples from hospital rooms and community isolation facilities

**DOI:** 10.1017/ice.2021.8

**Published:** 2021-01-25

**Authors:** Sean Wei Xiang Ong, Yian Kim Tan, Kristen Kelli Coleman, Boon Huan Tan, Yee-Sin Leo, Dong Ling Wang, Ching Ging Ng, Oon-Tek Ng, Michelle Su Yen Wong, Kalisvar Marimuthu

**Affiliations:** 1 National Centre for Infectious Diseases, Singapore; 2 Department of Infectious Diseases, Tan Tock Seng Hospital, Singapore; 3 DSO National Laboratories, Singapore; 4 Duke-NUS Medical School, National University of Singapore; 5 Yong Loo Lin School of Medicine, National University of Singapore, Singapore; 6 Lee Kong Chian School of Medicine, Nanyang Technological University, Singapore; 7 Infection Prevention and Control Office, Woodlands Health Campus, Singapore

**Keywords:** COVID-19, SARS-CoV-2, aerosols, airborne, viral culture, air sampling

## Abstract

**Background:**

Understanding the extent of aerosol-based transmission of severe acute respiratory syndrome coronavirus 2 (SARS-CoV-2) is important for tailoring interventions for control of the coronavirus disease 2019 (COVID-19) pandemic. Multiple studies have reported the detection of SARS-CoV-2 nucleic acid in air samples, but only one study has successfully recovered viable virus, although it is limited by its small sample size.

**Objective:**

We aimed to determine the extent of shedding of viable SARS-CoV-2 in respiratory aerosols from COVID-19 patients.

**Methods:**

In this observational air sampling study, air samples from airborne-infection isolation rooms (AIIRs) and a community isolation facility (CIF) housing COVID-19 patients were collected using a water vapor condensation method into liquid collection media. Samples were tested for presence of SARS-CoV-2 nucleic acid using quantitative real-time polymerase chain reaction (qRT-PCR), and qRT-PCR-positive samples were tested for viability using viral culture.

**Results:**

Samples from 6 (50%) of the 12 sampling cycles in hospital rooms were positive for SARS-CoV-2 RNA, including aerosols ranging from <1 µm to >4 µm in diameter. Of 9 samples from the CIF, 1 was positive via qRT-PCR. Viral RNA concentrations ranged from 179 to 2,738 ORF1ab gene copies per cubic meter of air. Virus cultures were negative after 4 blind passages.

**Conclusion:**

Although SARS-CoV-2 is readily captured in aerosols, virus culture remains challenging despite optimized sampling methodologies to preserve virus viability. Further studies on aerosol-based transmission and control of SARS-CoV-2 are needed.

Aerosol-based transmission of severe acute respiratory syndrome coronavirus 2 (SARS-CoV-2) and its overall contribution to the coronavirus disease 2019 (COVID-19) pandemic has been a subject of intense debate.^1,2^ Aerosol-based transmission is defined as transmission through inhalation of particles dispersed through the air as aerosols. Although multiple studies have reported the detection of SARS-CoV-2 nucleic acid in air samples collected from a variety of healthcare and community settings,^3–8^ only 3 of these studies attempted in vitro viral culture to characterize the infectivity of the airborne virus particles.^6–8^ Among the 3 air sampling studies where viral culture was attempted, 1 study using a water-vapor condensation collection method resulted in the successful isolation of infectious SARS-CoV-2.^8^ These infectious aerosols were collected in a hospital room occupied by 2 confirmed COVID-19 patients, and the viral genome sequences matched the sequence in a respiratory sample of 1 of the patients in the room.^8^ Although these data strongly support aerosol-based transmission of SARS-CoV-2, further studies are needed to demonstrate the reproducibility of these results across a variety of settings and patients.

We have previously described results of positive air samples in 2 hospital rooms housing COVID-19 patients early in the course of illness, though viral culture was not performed in that pilot study.^5^ Hence, as a follow-up to that study, we validated and adapted a water vapor condensation collection method similar to that reported by Lednicky et al^8^ to collect air samples from hospital rooms and community isolation facilities housing COVID-19 patients, and we evaluated quantitative real-time polymerase chain reaction (qRT-PCR) positive air samples for the presence of infectious SARS-CoV-2 through viral culture. We hypothesized that infectious SARS-CoV-2 could be isolated from air samples obtained from rooms of patients early in their illness, when viral shedding from the respiratory tract tends to peak.^9,10^

## Methods

### Study setting

This study was conducted in airborne-infection isolation rooms (AIIRs) at the National Centre of Infectious Diseases, Singapore; as well as a community isolation facility (CIF) housing confirmed COVID-19 patients not requiring inpatient care. The AIIRs were completely enclosed negative pressure rooms with 12 air changes per hour, and they housed either 1 or 2 COVID-19 patients each. Airflow direction was from the ceiling towards air vents located behind the patients’ beds, just above the ground. The size of these rooms was ˜8 m by ˜8 m by ˜2.5 m, with a total air volume of ˜160,000 L. Rooms housing patients within the first week of illness were preferentially selected because this is when viral shedding and infectivity is highest, and all patients were confirmed to have COVID-19 via SARS-CoV-2 PCR at the hospital laboratory. Clinical data were collected from medical records using a standardized data collection form.

The CIF was a large, naturally ventilated facility, which used to function as an exhibition center, with a capacity of housing 2,700 patients. The facility was not enclosed, with one side of the facility facing the sea, providing thorough ventilation. In accordance to Singapore public health policy, COVID-19 patients who did not require further inpatient care were transferred to a community isolation facility for continued isolation until at least 21 days after illness onset.^11^ This facility was divided into cubicles housing 10 patients each, separated by surrounding temporary walls from other cubicles, without ceilings or windows. Airflow direction was variable given the natural ventilation and was not measured. Clinical data could not be collected from the patients at this facility.

## Air sampling

**Fig. 1.**
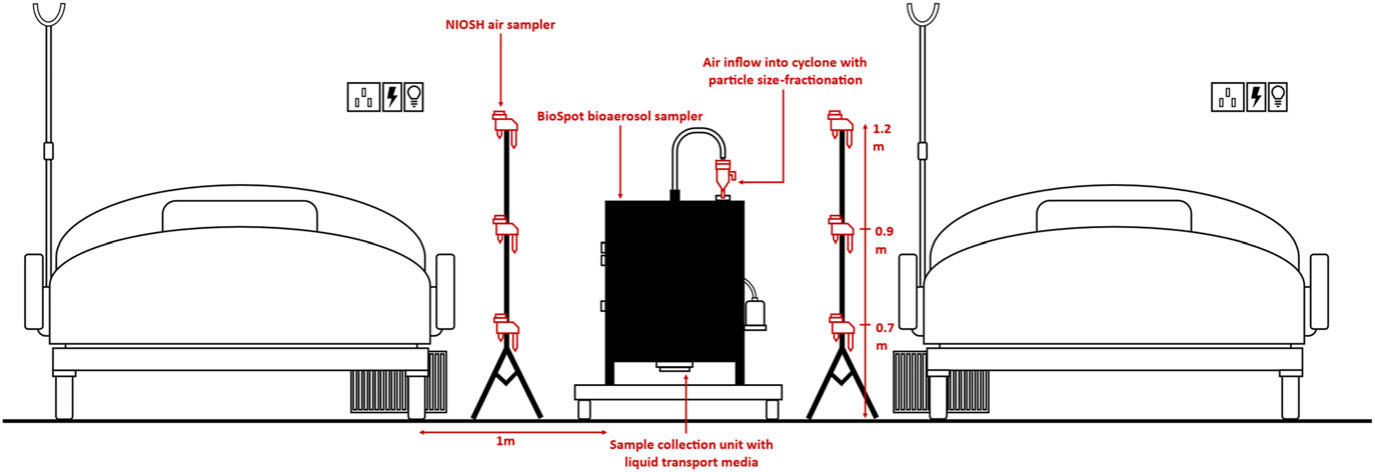
Air sampling set-up in hospital airborne-infection isolation room.

**Fig. 2.**
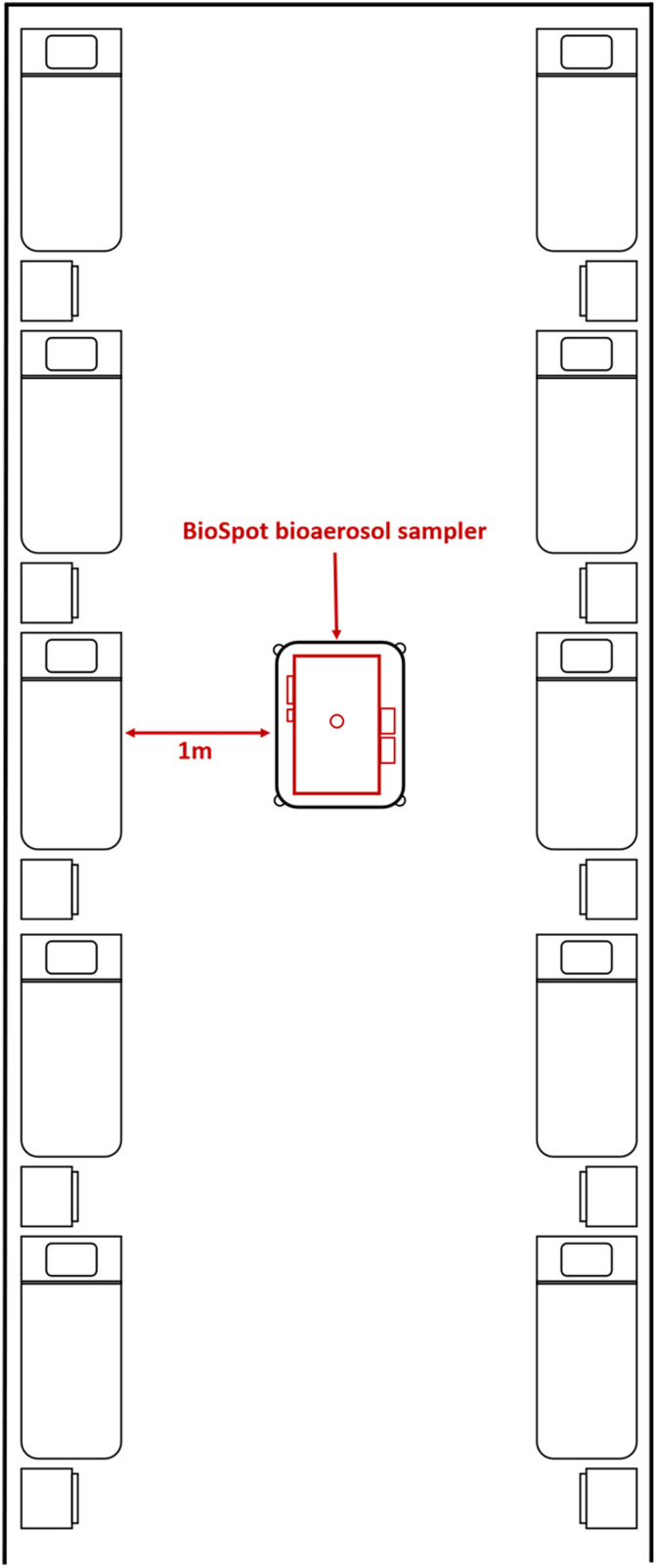
Air sampling set-up in community isolation facility cubicle.

Air samples were collected using a BioSpot-VIVAS BSS300-P bioaerosol sampler (Aerosol Devices, Fort Collins, CO), which collects airborne particles using a water-vapor condensation method into a liquid collection medium at a flow rate of 8 L per minute. This method has been previously used to successfully isolate other respiratory viruses such as influenza.^12,13^ A GK4.162 (RASCAL) cyclone (Mesa Laboratories, Butler, NJ) was affixed to the sampling inlet during selected sampling cycles to filter out particles >4.34 µm in diameter, selectively collecting only small particles <4.34 µm in size at the flow rate of 8 L per minute. The sampling inlet was placed at a height of 1 m and a distance of 1 m from the patients’ beds in both the AIIRs and the CIF. The air sampling configurations illustrating relative distances between the samplers and the patients in both the hospital AIIR and the CIF are shown in Figures 1 and 2, respectively.

To validate this sampling protocol, for the first 2 sampling cycles, 6 additional NIOSH BC-251 bioaerosol samplers (US National Institute for Occupational Safety and Health) connected to SKC AirChek TOUCH Pumps (SKC, Eighty-Four, PA) were used (methodology as previously described^5^) to ensure that results were concordant between the different sampling methods. Because results between both sampling methods were concordant for the first 2 sampling cycles, only the BioSpot sampler was used for subsequent sampling.

### Quantitative real-time polymerase chain reaction methods

Air samples were tested for the presence of SARS-CoV-2 via qRT-PCR. Sample RNA extraction was conducted using the QIAamp viral RNA mini kit (Qiagen, Hilden, Germany) according to the manufacturer’s instructions. Real-time PCR assays targeting the envelope (E) gene^14^ and orf1ab assay adapted from Drosten et al^15^ were used for the detection of SARS-CoV-2 RNA. For the E gene assay, 20 µL reaction mix was prepared with 12.5 µL SuperScript III Platinum One-Step qRT-PCR Kit (ThermoFisher Scientific, Waltham, MA, USA) buffer, 0.75 mM Mg_2_SO_4_, 5 µL RNA, 400 nM each of forward primer (E_Sarbeco_F1-ACAGGTACGTTAATAGTTAATAGCGT) and reverse primer (E_Sarbeco_R2-ATATTGCAGCAGTACGCACACA) with 200 nM probe (E_Sarbeco_P1-(FAM) ACACTAGCCATCCTTACTGCGCTTCG (BHQ1)). Thermal cycling conditions included reverse transcription at 55°C for 10 minutes, an initial denaturation at 95°C for 5 minutes, followed by 45 cycles of 95°C for 15 seconds, 58°C for 1 minute. For the orf1ab assay, 20 µL reaction mix was prepared with 12.5 µL SuperScript III Platinum One-Step qRT-PCR Kit buffer, 0.5 mM Mg_2_SO_4_, 5 µL RNA, 800 nM each of the orward primer (Wu-BNI-F-CTAACATGTTTATCACCCGCG) and reverse primer (Wu-BNI-R-CTCTAGTAGCATGACACCCCTC) with 400 nM probe (WU-BNI-P-(FAM) TAAGACATGTACGTGCATGGATTGGCTT (BHQ1)). Thermal cycling conditions included reverse transcription at 55°C for 10 minutes, an initial denaturation at 95°C for 5 minutes, followed by 45 cycles of 95°C for 15 seconds, 60°C for 1 minute. All samples were run in duplicate and with both assays. Positive detection was recorded as long as amplification was observed in at least 1 assay.

### Virus culture methods

PCR-positive aerosol samples collected by the BioSpot-VIVAS BSS300-P sampler were further evaluated for virus viability via cell culture. Monolayers of Vero C1008 cells (ATCC-1586) in T25 flasks were inoculated with 1 mL inoculum (500 µL of the swab sample and 500 µL Eagle’s MEM) and cultured at 37°C, 5% CO_2_ with blind passage every 7 days. Thereafter, 140 µL cell culture was used for RNA extraction and real-time PCR twice per week, to monitor changes in target SARS-CoV-2 genes as an indication of successful viral replication. In the absence of cytopathic effects and real-time PCR indication of viral replication, blind passages continued for a total of 4 passages before any sample was determined to be negative of viable SARS-CoV-2 virus particles.

Informed consent was waived as there was no direct interaction with the patients. Clinical data were collected as part of a separate retrospective cohort study of COVID-19 patients (National Healthcare Group Domain Specific Review Board, reference no. 2020/01122).

## Results

### Setting and patient selection

In total, 12 sampling cycles were carried out in hospital AIIRs, 8 in rooms housing 2 patients and 4 in rooms housing 1 patient (Table 1). One room was sampled twice 48 hours apart; hence, the total number of unique patients involved was 19. 18 patients (94.7%) were male, and median age was 43 years (interquartile range [IQR], 34–48). The median day of illness was day 5 (IQR, 4–7), and 12 (63.2%) patients were symptomatic on the day of sampling. None of the patients needed supplemental oxygen or underwent aerosol-generating procedures in the 24 hours preceding sampling, and none was critically ill, intubated, or on mechanical ventilation. Also, 9 sampling cycles were carried out in the CIF cubicles, with 10 patients in each cubicle at the time of sampling. In this patient group, day of illness could not be determined, but air sampling was performed within 7 days of a PCR-positive clinical swab finding. Formal clinical data collection from CIF patients was not permitted due to lack of time to obtain institutional board review approval for clinical data collection at this external site while COVID-19 patients were available for study.

**Table 1.** Clinical Characteristics of COVID-19 Patients and Corresponding Air Sampling Results From Their Hospital Airborne-Infection Isolation Rooms

Sampling Cycle	Patient Number	Age	Sex	Day of Illness	Symptoms on Day of Sampling	Clinical Ct Value^a^	Cyclone Filtering Particles <4.34 µm	SARS-CoV-2 RNA^b^ Copies per m^−3^ Air	Air Sample Ct Value	Virus Culture Result
1^c^	1	45	M	3	Fever	23.33	Yes	ND	…	…
	2	43	M	4	Nil	32.47				
2^c^	3	54	M	5	Cough	19.61	Yes	469.9	36.06	Negative
	4	26	M	4	Nil	15.22				
3	5	43	M	5	Fever	16.76	Yes	1431.6	35.02	Negative
	6	47	M	6	Nil	NA				
4	7	46	M	5	Nil	NA	Yes	ND	…	…
	8	51	M	6	Nil	NA				
5	9	41	M	10	Nil	NA	Yes	ND	…	…
	10	48	M	8	Cough	NA				
6	11	48	M	11	Fever	NA	Yes	ND	…	…
	12	24	M	3	Fever	24.05				
7	13	35	M	5	Sore throat, cough	NA	No	2738.4	32.62	Negative
	14	37	M	8	Nausea, anorexia	NA				
8	15	50	M	3	Fever, cough	15.99	No	385.7	37.27	Negative
9	15	50	M	5	Cough, coryza		Yes	217.9	35.89	Negative
10	16	34	M	5	Nil	20.77	Yes	ND	…	…
11	17	21	M	4	Sore throat	20.53	Yes	178.9	38.36	Negative
	18	26	M	7	Sore throat	17.50				
12	19	37	F	3	Headache	25.02	Yes	ND	…	…

Note. ND, none detected; Ct, cycle threshold; NA, not available.

a PCR cycle threshold value from patient’s respiratory sample collected within 72 hours prior to room air sampling. PCR target using E or N2 gene.

b ORF1ab gene copies.

c NIOSH aerosol samples were collected from rooms 1 and 2 for BioSpot sampler validation. Results were agreeable (see Table 2).

### Air samples

Of 12 BioSpot air samples from hospital AIIRs, 6 (50%) were positive for SARS-CoV-2 nucleic acid. Among these positive air samples, concentrations of virus copies per cubic meter of air ranged from 178.9 to 2,738.4 (using the ORF1ab gene target for calculation as this target was consistently detected across all positive samples). Of these, 4 samples were size fractionated to contain only particles <4.34 µm in diameter, while 2 were not size fractionated. All positive samples were from rooms with at least 1 symptomatic patient, and all patients were early in the illness course (within seven days). For the first 2 sampling cycles, NIOSH aerosol samplers were also used to collect aerosols in the rooms to validate the BioSpot sampling method (Fig. 1). Results were agreeable (Table 2), and SARS-CoV-2 nucleic acid was detected in aerosols <1 µm, 1–4 µm, and >4 µm in diameter.

**Table 2.** NIOSH and BioSpot Aerosol Samples Collected From Double-Occupancy Airborne-Infection Isolation Rooms of COVID-19 Patients

Room	No. of Patients	Day of Illness	Clinical Ct Values^a^	SARS-CoV-2 RNA Copies per m^–3^ Air	Aerosol Particle Size	Sampler
1	2	3; 4	23; 32.5	ND ND	<1 μm, 1–4 μm, >4 μm, <4.34 µm	NIOSH^b^ BioSpot^c^
2	2	5; 4	19.6; 15	40.48 908.02	<1 μm 1-4 μm	NIOSH^b^
				1,083.1 469.9	>4 μm <4.34 µm	BioSpot^c^

Note. ND, none detected; PCR, polymerase chain reaction; Ct, cycle threshold; NIOSH, National Institute for Occupational Safety and Health.

a PCR cycle threshold values from patient respiratory samples collected within 72 h of room sampling.

b Six 840-L samples pooled and analyzed together (5,040 L air total). Size fractionation retained.

c One 3,840-L sample.

Only 1 (11.1%) of 9 samples from the CIF was positive for SARS-CoV-2 nucleic acid, with a concentration of 978.3 ORF1ab gene copies per cubic meter of air. This was a non–size-fractionated sample. The other 4 size-fractionated samples and 4 non–size-fractionated samples were negative via qRT-PCR. Virus cultures of all 7 qRT-PCR–positive BioSpot air samples were negative after 4 blind passages.

## Discussion

In this air sampling study conducted in hospital rooms and a community isolation facility, air samples collected from the environments of COVID-19 patients were frequently positive for SARS-CoV-2 nucleic acid via PCR, though PCR-positive air samples were negative on viral culture. Although SARS-CoV-2 nucleic acid has been frequently detected in air samples, viable virus isolation from the air has been reported by only 1 study.^8^ Lednicky et al^8^ described the isolation of viable virus in from air samples collected from 1 hospital room housing 2 COVID-19 patients. Although these data support aerosol-based transmission, they are limited by their small sample size. More studies including larger samples sizes or longitudinal cohorts are needed to accurately measure the amount of viable virus in aerosols emitted by COVID-19 patients.

It is well understood that viral shedding from the respiratory tract of COVID-19 patients tends to peak early in the disease course,^9,10^ and results from both our current study and previous pilot study demonstrate our ability to capture aerosolized SARS-CoV-2 from nearby patients with known clinical cycle threshold (Ct) values below 21.^5^ Taken together, it is plausible to estimate that the risk of SARS-CoV-2 infection through inhalation is lower when COVID-19 patients are later in their illness and have higher clinical Ct values and SARS-CoV-2 in nearby aerosols is below the detection limit or is not present. But again, measurements of infectious virus emission rates across a variety of patients are needed to more accurately assess this risk.

Size fractionation of aerosols containing infectious virus is also an important component of measuring risk of infection because the size of a virus-laden aerosol is indicative of where in the respiratory tract it can be deposited and the type of infection or immune response that might ensue. Aerosol size fractionation was not performed by Lednicky et al^8^; thus, the amount of infectious SARS-CoV-2 carried in respirable aerosols (<5 µm in diameter) is unknown. We attempted to address this knowledge gap by performing aerosol size fractionation in our study, and although SARS-CoV-2 RNA was detected in respirable aerosols, virus cultures were negative. Notably, we detected SARS-CoV-2 RNA in aerosols <1 µm in diameter, which we failed to accomplish in our previous study.^5^

In vitro cell culture is considered the gold standard method for determining virus infectivity, but technical limitations to this approach must be considered when studying environmental samples containing low virus concentrations when compared to human clinical samples. Successfully isolating infectious virus from air samples is known to be challenging due to the degradation of viral material during the collection process.^16^ To increase the probability of viable virus recovery from air samples collected in this study, we used a similar water-vapor condensation method and bioaerosol collector as described by Lednicky et al.^8^ The BioSpot collection device used is designed to mimic the physiological conditions of the human lungs, to better preserve pathogen viability, which is often compromised when using dry cyclone air sampling devices. Although a significant proportion of air samples in our study were positive for SARS-CoV-2 via qRT-PCR, all virus cultures were negative despite optimization of the sampling methodology. However, the failure to isolate viable virus from the air does not necessarily mean that patients are not shedding infectious aerosols. Numerous factors may compromise successful virus isolation, such as sample collection media, sample transfer, sample processing, and in vitro cell culture infection method, which may be enhanced by using engineered cell lines. For example, Vero E6 cells expressing TMPRSS2 have been demonstrated to enhance SARS-CoV-2 isolation.^17^ For viral culture in this study, we used standard Vero E6/C1008 cells. Such technical and virological caveats should be considered when interpreting air sampling data.

Our study has several further limitations. Although our sample size was larger than earlier pilot studies performed by our group and other authors, the generalizability of our findings is still limited for several reasons. First, we preferentially selected patients early in their illness course and with a lower Ct value because we hypothesized this would maximize the possibility of successfully isolating viable virus. Second, most of our patients had only mild disease, without requiring supplemental oxygen, and our results may have varied if we sampled patients on high-flow oxygen or in the intensive care unit. Thirdly, sampling was conducted in a naturally ventilated community isolation facility, and airborne-infecition isolation hospital rooms (designed to limit transmission of airborne infections). Each of these settings has a different heating, ventilation, and air conditioning (HVAC) system compared to other community venues (eg, schools and bars), thus limiting the generalizability of our findings to transmission in the community, which is where bulk of SARS-CoV-2 transmission occurs.

For example, superspreading events such as those in choirs, churches, or restaurants seem to be best explained by aerosol-based transmission.^18–20^ However, these events appear to be sporadic, and the secondary attack rates among household or close contacts in other transmission studies are reported to be lower than what would be expected for a virus classically classified as airborne,^21–23^ indicating that SARS-CoV-2 transmission dynamics display significant heterogeneity.^24^ Furthermore, large cohort studies have demonstrated that a minority of infectious individuals account for a disproportionate number of secondary cases.^25^ Although this further complicates our collective understanding of how and by whom SARS-CoV-2 is readily transmitted, it can partly explain the sporadic nature of SARS-CoV-2 superspreading events.

Aerosol-based transmission of SARS-CoV-2 appears to be occurring, and knowledge gaps remain regarding its overall contribution to the COVID-19 pandemic. More information is needed on inhalation dose and patient factors that influence shedding of infectious aerosols. Follow-up air sampling studies with a larger sample size across a wider range of patients and healthcare and community settings are needed.
